# Molecular medicine tumor board: whole-genome sequencing to inform on personalized medicine for a man with advanced prostate cancer

**DOI:** 10.1038/s41391-021-00324-5

**Published:** 2021-02-10

**Authors:** Andrew J. Armstrong, Xiaotong Li, Matthew Tucker, Shantao Li, Xinmeng Jasmine Mu, Kenneth Wha Eng, Andrea Sboner, Mark Rubin, Mark Gerstein

**Affiliations:** 1 Duke Cancer Institute Center for Prostate and Urologic Cancer, Departments of Medicine, Surgery, Pharmacology and Cancer Biology, Duke Cancer Institute, Durham, NC USA; 2 Program in Computational Biology and Bioinformatics, Yale University, New Haven, CT USA; 3 Department of Medicine, Vanderbilt University Medical Center, Nashville, TN USA; 4 The Broad Institute of MIT & Harvard, Cambridge, MA USA; 5 Department of Physiology and Biophysics, Englander Institute for Precision Medicine, HRH Prince Alwaleed Bin Talal Bin Abdulaziz Alsaud Institute for Computational Biomedicine, Weill Cornell Medical College, New York, NY USA; 6 Department of Pathology and Laboratory Medicine, Englander Institute for Precision Medicine, HRH Prince Alwaleed Bin Talal Bin Abdulaziz Alsaud Institute for Computational Biomedicine, Meyer Cancer Center, Weill Cornell Medical College, New York, NY USA; 7 Department for BioMedical Research, University of Bern and Inselspital, 3010 Bern, Switzerland; 8 Bern Center for Precision Medicine, University of Bern and Inselspital, 3010 Bern, Switzerland; 9 Department of Molecular Biophysics and Biochemistry, Department of Statistics and Data Science, Department of Computer Science, Yale University, New Haven, CT USA

**Keywords:** Cancer genetics, Translational research

## Abstract

**Purpose:**

Molecular profiling of cancer is increasingly common as part of routine care in oncology, and germline and somatic profiling may provide insights and actionable targets for men with metastatic prostate cancer. However, all reported cases are of deidentified individuals without full medical and genomic data available in the public domain.

**Patient and methods:**

We present a case of whole-genome tumor and germline sequencing in a patient with advanced prostate cancer, who has agreed to make his genomic and clinical data publicly available.

**Results:**

We describe an 84-year-old Caucasian male with a Gleason 10 oligometastastic hormone-sensitive prostate cancer. Whole-genome sequencing provided insights into his tumor’s underlying mutational processes and the development of an *SPOP* mutation. It also revealed an androgen-receptor dependency of his cancer which was reflected in his durable response to radiation and hormonal therapy. Potentially actionable genomic lesions in the tumor were identified through a personalized medicine approach for potential future therapy, but at the moment, he remains in remission, illustrating the hormonal sensitivity of his *SPOP*-driven prostate cancer. We also placed this patient in the context of a large prostate-cancer cohort from the PCAWG (Pan-cancer Analysis of Whole Genomes) group. In this comparison, the patient’s cancer appears typical in terms of the number and type of somatic mutations, but it has a somewhat larger contribution from the mutational process associated with aging.

**Conclusion:**

We combined the expertise of medical oncology and genomics approaches to develop a molecular tumor board to integrate the care and study of this patient, who continues to have an outstanding response to his combined modality treatment. This identifiable case potentially helps overcome barriers to clinical and genomic data sharing.

## Introduction

Over the last 20 years, the genomic heterogeneity of prostate cancer (PC) has become increasingly recognized across the spectrum of localized and metastatic disease [1–5]. Emerging data support that PC is driven by a number of genetic variants, including specific gene fusions, gains and losses of chromosomal regions, point mutations, and variations in epigenetic signatures (e.g., histone methylation). These subgroups may provide additional prognostic stratification. For example, with combined genomic/epigenomic subgrouping of PC data from the Cancer Genome Atlas, nearly three-fourths of all PC cases could be categorized into one of four gene fusion groups (*ERG, ETV1, ETV4*, and *FLI1*) or three gene mutation groups (*SPOP, FOXA1*, and *IDH1*) [4]. Moreover, genomic subtypes including luminal and basal classifications can be differentiated based on clinical outcomes [6].

In the clinic, men with metastatic PC frequently harbor germline or somatic defects in traditional DNA repair genes such as *BRCA1, BRCA2, ATM*, and *FANCD2*, with ~20% of cases harboring such deficiencies [2, 4, 7]. Therefore, national guidelines now recommend germline testing for all high-risk and metastatic patients, and for those with a family history suggestive of cancer. Such alterations in DNA repair enzymes may impact familial risk and genetic counseling as well as opportunities for therapy, such as recently FDA-approved poly ADP-ribose polymerase inhibitors for tumors with known DNA repair defects or pembrolizumab for men with microsatellite-high and/or mismatch repair-deficient tumors [8, 9].

Many centers are increasingly using targeted next-generation sequencing panels which include many of the most common mutations including DNA repair and mismatch repair enzymes as well as somatic biallelic *CDK12* inactivation, a newly described immunogenic subset of prostate cancer [10–12]. However, with falling price of sequencing and the possibility of identifying novel genomic events, whole-genome approaches may provide additional clinical benefits beyond targeted sequencing.

Here, we provide a case report describing the diagnosis and treatment of a man with primary M1 oligometastatic PC who volunteered to release his entire protected health information, including the results of whole-genome sequencing (WGS) of his germline and both whole-exome sequencing (WES) and WGS of his PC. Through a multi-institutional clinical-molecular tumor board, we integrated clinical, somatic, and germline genomic sequencing data, and used bioinformatics to put his tumor in the context of (1) existing knowledge of PC biology, (2) his outstanding response to combined radiation and hormonal therapy, and (3) future clinical management recommendations. This case study demonstrates the utility of translational genomics in cancer precision medicine.

## Patient and methods

After diagnosis, the patient provided informed consent and underwent WES and WGS of both tumor tissue (tumor content = 66.9%) and normal blood samples. For WES, the tumor was sequenced to an average coverage of 104x using an Agilent HaloPlex, covering 21,522 genes. Sequencing was performed using the Illumina HiSeq 2500 platform. Sequencing reads were aligned to the GRC37/hg19 reference and processed according to the WES Test for Cancer-ExaCT1-pipeline v0.9 with a capture efficiency of 90.29% [13]. For WGS, the average sequence depth was ~120X for the subject and ~60X for the matched normal. We took the intersection of somatic variants call sets generated by MuTect [14] and Strelka [15]. Using all sequencing data, we analyzed the subject’s germline and somatic mutations in comparison with a cohort of WGS PC cases [16]. We processed the germline WGS data using a standard pipeline following GATK [17] best practices. The patient also provided reports from a commercial Hereditary Cancer Risk Test (color assay on saliva; *n* = 30 genes), and a HIPAA waiver for disclosure of his personal and genomic data. Computer code used to generate the results in this study is available from the corresponding author on reasonable request.

## Results

At the time of initial presentation in 2015, the patient was 79 years old and was being followed closely by his primary care provider for increasing lower urinary tract symptoms, including increased frequency, urgency, nocturia, and weakened stream. The patient is of Northern European ancestry and had a positive family history for PC in a cousin (non-lethal, age 68) and paternal uncle, and multiple family members with lung cancer (daughter, sister, mother, son). He was a former smoker (10 pack-years) and retired investment banker and economist without other significant exposures. He had been followed with yearly prostate specific antigen (PSA) levels since age 50 in the normal range (<4.0 ng/mL) and with normal digital rectal examinations, but repeat PSA testing demonstrated a dramatic increase to 13.08 ng/mL on 1/19/2015 with digital rectal exam notable for an indurated prostate with bilateral nodularity measuring ~50 cc volume (cT2 stage).

**Fig. 1:**
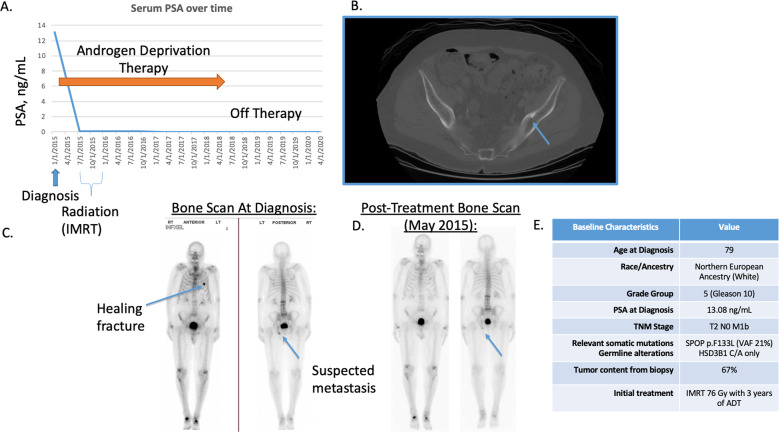
Summary of the patient case and outcomes. **A** Response of serum PSA to initial therapy with IMRT and 3 years of ADT, with ongoing response off therapy now for 2 years. **B** Staging CT at diagnosis demonstrating a left pelvic/ischia osteoblastic metastasis, confirmed on **C** bone scan at baseline/diagnosis in 2015 and **D** subsequent to therapy 3 months after treatment initiation, showing a favorable treatment effect. **E** Summary table of our patient’s case report.

The patient underwent transrectal ultrasound-guided biopsy on 2/10/2015, revealing a bilateral Gleason 5 + 5 = 10 adenocarcinoma (Grade Group 5) with 12/12 cores positive for high-volume, high-risk disease, including regions of Gleason 5 + 3 = 8 and 4 + 5 = 9 adenocarcinoma, without small-cell features. Perineural invasion was noted diffusely. Staging bone scintigraphy using ^99m^Tc with methylene diphosphonate showed a focus of increased radiotracer activity in the left ischium, which correlated with computerized tomography (CT) imaging, consistent with osseous metastatic disease (Fig. 1). Computerized tomography imaging showed no evidence of lymphadenopathy or visceral metastatic disease. As such, the patient was staged as T2bNXM1 PC and began androgen deprivation therapy (ADT) on 2/12/2015 with once-daily bicaluatmide 50 mg for 30 days along with a triptorelin pamoate 22.5 mg depot injection.

On 5/11/2015, the patient’s repeat PSA was down to 0.09 ng/mL (Fig. 1A) and repeat bone scintigraphy showed decreased radiotracer activity in the left ischium and no new evidence of metastatic disease (Fig. 1B–D). His PSA increased slightly to 0.11 ng/mL on 7/21/2015 and he subsequently underwent transrectal ultrasound-guided fiducial marker placement, along with 38 fractions of intensity-modulated radiation therapy (IMRT) to his prostate and seminal vesicles. From 8/18/2015 to 10/2/2015, he received 15 fractions of IMRT boost to his prostate for a total of 7600 centigray. His ADT was switched to leuprolide acetate 45 mg intramuscularly and was continued every six months. No pelvic or metastatic site radiation was performed. One year after diagnosis (1/25/2016), the patient’s PSA levels were 0.05 ng/mL. From September 2016 through September 2018 he was maintained on ADT and his PSA remained undetectable. His ADT was held in October 2018 and his PSA has remained 0.01–0.02 through his most recent checkup on 4/1/2020 (Fig. 1). Overall, the patient has tolerated ADT well with minimal adverse effects that include weight gain, intermittent hot flashes, and decreased libido. He began intermittent ADT in 2018 and presently remains off therapy through November 2020 and free of detectable disease at the age of 83 with a PSA of 0.01. Adverse effects from radiation were also minimal and consisted primarily of intermittent diarrhea that resolved. Adverse effects from hormonal therapy have included weight gain, mild to moderate fatigue, muscle loss, and mild hot flashes. The patient’s summary clinical data is provided in Fig. 1E.

Throughout the course of treatment, the patient has maintained a healthy lifestyle with regular exercise, emphasizing restorative yoga, and a predominately vegan diet. He does not drink alcohol and has a remote five-year history of cigarette smoking, having quit in 1964. He studied system theory, mathematics, and predictive algorithms in economics and epistemology and has been very involved in his treatment decisions and genomic analyses.

After providing informed consent, the patient underwent WES and WGS somatic and germline analysis at Weill Cornell Medical College as part of a Precision Medicine research study, in conjunction with clinical care at the Duke Cancer Center and Duke Cardiology. Integrated bioinformatics analysis was performed at Yale University.

The patient first underwent standard-of-care germline testing using a 30-gene-panel assay for hereditary DNA repair defects and other pathogenic clinical mutations associated with hereditary PC or unfavorable clinical outcomes [7]. Using a commercial Hereditary Cancer Risk Test on 12/31/16, no mutations were identified in 31 DNA homologous or mismatch repair genes, including *BRCA1/2, ATM, POLD1/POLE, RAD51D, TP53, NBN, PALB2, PMS2, MLH1, MSH2, MSH6, MYTYH*, and *EpCAM*. In addition, immunohistochemistry analysis of his prostate biopsy did not reveal loss of *MLH1, MSH2, MSH6*, or *PMS2*, and his tumor was found to be microsatellite stable.

**Fig. 2:**
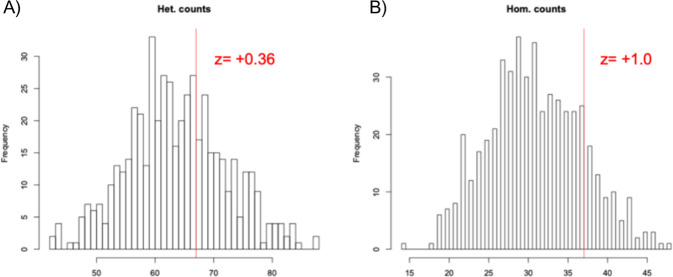
Enrichment of GWAS risk alleles. **A** The number of heterogenous states of risk alleles (i.e., carry one risk allele) in an individual. **B** The number of homogenous states of GWAS risk alleles (i.e., carry two risk alleles) in an individual. In both cases, our subject carries slightly higher risk alleles (z-score: +0.36 and +1.0 respectively).

On 6/21/2016, the patient’s prostate biopsies were subjected to next-generation WES and WGS with a matched normal control at Weill Cornell Medical College. Germline WGS data further confirmed that the subject did not carry any significant germline mutations in DNA repair genes. In a panel of genome-wide association study (GWAS) risk alleles (*N* = 428), his genome demonstrated insignificant enrichment for risk alleles when compared to 503 genomes of European ancestry (EUR) from the 1000 Genomes Project [18], carrying 67 heterozygous risk alleles (average in EUR: 64; z = +0.36) (Fig. 2A) and 37 homozygous risk alleles (average in EUR: 31; z = 1.0) (Fig. 2B). Additionally, the subject does not carry the *HOXB13* G84E variant, which is associated with a significantly higher risk of hereditary PC [19].

Studies have shown that a germline missense variation (rs1047303) in *HSD3B1* is predictive for ADT failure [20–22], especially in individuals with homozygous risk alleles (C/C). WGS revealed that our patient is heterozygous (C/A) for this single-nucleotide polymorphism (SNP), suggesting a prolonged and more robust response to ADT. Somatic loss of heterozygosity of this variant has been reported to be a significant event in PC; however, we did not find evidence for this in his tumor sample (frequency of allele = 0.508 in germline, 0.659 in somatic). Recent evidence suggests that individuals with rs1047303 might metabolize abiraterone differently and this may be associated with androgen receptor (AR) agonism; thus, the clinical activity of abiraterone acetate is uncertain should he continue with ADT, and alternatives such as enzalutamide may be considered [23].

Using WGS, we found that the subject carries 4780 SNPs (z-score = +0.47, log-transformed) and 332 insertions/deletions. We further stratified the SNPs into 58 coding mutations (z-score after normalized by mutation load = −0.73) and 66 high-functional-impact noncoding mutations (FunSeq [24] score>1.5; z-score after normalized by mutation load = +1.11). The Variants Effect Predictor [25] found 0 high-impact and 44 moderate-impact mutations (z-score after normalizing by mutation load = −1.02 and +0.53, respectively).

**Fig. 3:**
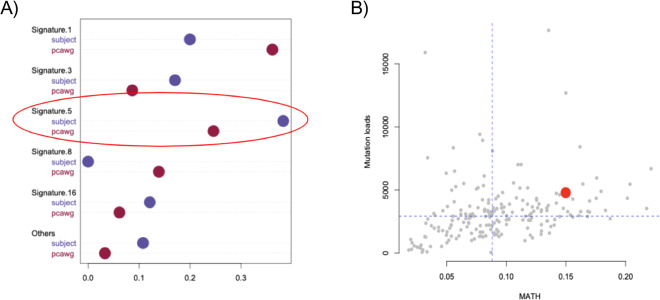
Somatic mutatioal signatures and tumor heterogeneity. **A** A dotchart showing the fractions of activities of signatures in the subject (blue) and average PCAWG individuals (red). The subject has higher signature 5. **B** The subject (red) when compared to the PCAWG individuals (gray), has slightly higher mutation load (*y*-axis) and higher MATH score (*x*-axis).

Using sigLASSO [26] and 30 COSMIC [27] signatures, we next examined somatic mutational patterns. The subject showed a large amount of activity from signature 5 (Fig. 3A). This signature has been demonstrated to be simply associated with age [28], consistent with the subject’s advanced age (81 versus 58 in the Pan-Cancer Analysis of Whole Genomes (PCAWG) cohort [29]). The patient showed a low percentage of signature 1, with characteristic spikes associated with C > T mutations in CpGs, indicating reduced genome methylation. Indeed, when comparing the percentage of C > T mutations in CpGs, our patient’s tumor ranks in the lower quartile within the published cohort (rank: 31/200, z-score = −1.06). We did not find other significant somatic mutational processes such as homologous repair deficiency or ApoBEC. We also calculated the mutant-allele tumor heterogeneity (MATH) score [30] using somatic mutations. In comparison with the PCAWG cohort, the subject showed a slightly higher score, which could indicate greater tumor heterogeneity. However, we suspect that this result is largely due to the patient’s higher mutation load (Fig. 3B).

**Fig. 4:**
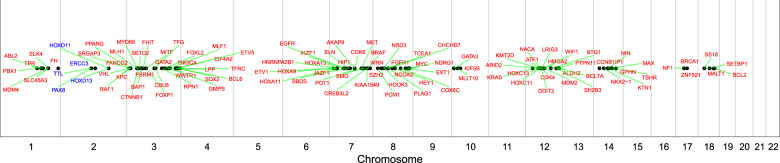
Cancer genes with copy number alterations. Cancer genes reporting genomic alterations identified from the subject were listed based on their locations on chromosomes. Genes in red had copy number gain events. Genes in blue reported copy number loss events.

In the targeted gene panel, we sequenced 59 clinically relevant genes linked to FDA-approved therapies and did not identify any actionable alterations. Additionally, we evaluated 575 known cancer genes using the WES results, and identified 20 alterations (Fig. 4, Supplementary Table 1). Finally, we identified 18 genes with point mutations or insertions/deletions with unknown clinical significance (Supplementary Table 2), including a missense mutation in the speckle-type pox virus and zinc finger protein *(SPOP)* gene (p.F133L) with a tumor variant allele frequency of 20.6%. Notably, this amino-acid change is highly recurrent in a large panel of PC samples [31] (3.6%, 36/1,013). The patient does not have a germline or somatic variant of rs1376350, a SNP at 7p14.3 that can associate with the *SPOP* mutation [32].

Although we did not identify additional common PC-driver mutations (e.g., *AR, RB1, PTEN, MYC*, or *TP53*), we did identify copy number alterations (Fig. 4, Supplementary Table 1). For example, we found a broad copy gain (likely trisomy) of chromosome 7 and 8 encompassing *MET, EZH2, EGFR*, and *MYC*; a broad copy gain of chromosome 3q encompassing the *SOX2* and *PIK3CA* loci; gain of 3p and the *FGFR1* locus; and copy losses at 2q encompassing the *PAX8* and *ERCC3* loci as well as the *HOXD11* and *HOXD13* loci. We did not find evidence of *CHD1* loss, which often co-occurs with *SPOP* mutations in PC. We observed deletions disrupting the estrogen receptor *ESR1* on chromosome 6. Multiple structural variant discovery tools have reported interchromosomal translocation and inversion events in *ESR1*, however the clinical significance is unknown.

Finally, we investigated the mutational burden on the entire genome with regards to epigenetics. To estimate the mutational burden, we tabulated the number of mutations observed in functional genomic regions as defined by epigenetic markers. We used ChromHMM [33] to segment the genome into 15 basic epigenetic states (e.g., enhancer, promoter) based on five histone modification signals. This is the first ChromHMM segmentation reported for primary prostate tissue. Next, we tallied the number of mutations of the subject and 199 PCAWG samples in each state. We found no significant differences between the subject and the PCAWG cohort in mutational burden (absolute z-scores < 2 for all 15 states).

## Discussion

### Significance of *SPOP* mutations

Here, we provide the first identifiable molecular tumor board case report of a patient with advanced PC. Our analysis includes comprehensive germline and somatic tumor whole-genome profiling as well as consideration of clinical outcomes. We found that this patient harbored oligometastatic hormone-sensitive PC driven by an age-related mutational process and higher-risk germline SNPs, leading to a pathogenic *SPOP* mutation. The patient is presently responding well to intermittent ADT and is expected to respond well based on the AR dependence of *SPOP*-mutant PC and his heterozygous *HSD3B1* gene. Beyond the genomic findings, this case report is unique as it details the patient’s goals, identity, medical history, preferences, and clinical outcomes.

While we did not identify any currently actionable mutations, the *SPOP* mutation has the potential to be actionable in the near future. *SPOP* is an E3-ubiquitin ligase with several substrates including the AR, and mutations disrupt proteosomal degradation, accumulation, and AR activity [34, 35]. Non-synonymous mutations in *SPOP* such as the F133L mutation in our patient are found in about 10% of PC cases [36]. The F133L alteration suggests an AR-dependent tumor, consistent with the patient’s prolonged clinical response to standard ADT. This alteration is enriched in localized-PC and de-enriched in mCRPC datasets [37, 38], suggesting that *SPOP* mutations select for hormonally responsive tumors with excellent outcomes.

Studies have shown that the SPOP enzyme is directly involved in homologous DNA repair. In vitro models knocking out *SPOP* demonstrate suboptimal DNA repair as well as increased sensitivity to ionizing radiation [35, 39]. Furthermore, studies using both mouse- and human-derived PC cell lines have shown that mutant *SPOP* (including *SPOP*-F133V) in concert with *CHD1* loss exhibits increased double-strand break repair and sensitivity to DNA-damaging agents such as poly ADP-ribose polymerase inhibitors [40]. Recent multicenter data from men with mHSPC treated with ADT suggest that SPOP mutations confer a favorable long-term prognosis on progression free and overall survival, which aligns with the current excellent outcome of our current patient [41].

### Analyses of the whole genome

In addition to *SPOP*, other cancer driver genes are altered in the subject’s tumor genome (Supplementary Table 3). Previous studies have shown that a germline missense variation (rs1047303) in the *HSD3B1* gene is predictive for ADT failure [20–22], especially in individuals with homozygous risk alleles (C/C). Based on the patient’s heterozygous *HSD3B1* SNP, his response and metabolism of abiraterone with resultant AR agonism might be of some concern; thus, enzalutamide, apalutamide, or darolutamide may be preferred over abiraterone acetate should the disease progress [23].

MET activation promotes tumor growth and metastasis. Therefore, inhibiting *MET* has been attractive therapeutically in several cancer types [42]. In PC, this therapy did not extend survival in Phase 3 clinical trials of unselected men. However, this trial was conducted in men heavily pretreated with mCRPC who harbored tumors that were likely highly heterogeneous with a high burden of metastases [43]. Cabozantinib may have greater activity in *MET*-amplified tumors, and its utility is unknown in earlier lines of therapy. Other oncologic processes of PC are being actively investigated. Genes regulating the epigenome and metabolic pathways have shown tremendous therapeutic potential [44]. In addition, although PC is considered an immunologically ‘cold’ tumor, active research and clinical trials aim to induce an immune response to PC, thereby turning ‘cold’ tumors ‘hot’. These advances present many potential treatment opportunities for PC patients.

With rich mutational information from WGS, we also explored the mutational landscape of the sample. We examined noncoding regions and explored mutational signatures, regulatory region mutational burden, and tumor heterogeneity. We determined that the underlying mutational process was most likely related to an age-related signature (signature 5), rather than a hereditary DNA repair deficiency or carcinogenic/exposure signature [28].

### Publicly available data

Our subject agreed to release all of his genomic sequencing data for public use, which has been deposited into the European Genome-Phenome Archive (EGAS00001004648). As described, this case is an elderly Caucasian man with high-risk bone oligometastatic PC, harboring a classic *SPOP* mutation, who has been well managed by IMRT and ADT and is presently enjoying a prolonged treatment-free interval. Our patient underwent a range of personal genomics tests, including direct-to-consumer commercial genetic tests as well as standard medical diagnostic WES and WGS on both germline and somatic cancer tissues over several years. These results form a rich genomic dataset of various quality, coverage, and resolution, and provide a unique opportunity to study how genomic sequencing operates in the real world. Importantly, we processed and provided rich annotations on the sequencing data, such as mutational patterns and signatures and epigenomic impacts, and compared his mutational profiles with 199 WGS PC samples from the PCAWG and other public datasets. In the era of precision medicine and accessible genome sequencing, we believe this will be a very useful data source for research and teaching purposes.

## Supplementary information

Supplementary Table 1

Supplementary Table 2

Supplementary Table 3

## Data Availability

Our subject agreed to release all of his genomic sequencing data for public use, which has been deposited into the European Genome-Phenome Archive (https://ega-archive.org; EGAS00001004648).
