# Supplementary material for: Molecular medicine tumor board: whole-genome sequencing to inform on personalized medicine for a man with advanced prostate cancer
Source: Prostate Cancer Prostatic Dis. 2021 Feb 10;24(3):786–93. doi: 10.1038/s41391-021-00324-5 (PMC8384621; doi:10.1038/s41391-021-00324-5)
Supplement: Supplementary file 1 — Supplementary Table 1 [file 41391_2021_324_MOESM1_ESM.pdf]

Altered region	Classification of CNA	Number of cancer genes	Cancer genes
1:162,729,675-205,687,469 q23.3-q32.1	broad copy number gain	6	MDM4, TPR, ELK4, PBX1, ABL2, SLC45A3
1:241,667,441-248,845,135 q43-q44	broad copy number gain; partial	1	FH
10:32,101,688-33,484,460 p11.22	focal amplification	1	KIF5B
10:93,416-27,284,037 p12.1-p15.3	broad copy number gain	2	GATA3, MLLT10
12:21,531,263-132,632,632 p11.1-q24.33	broad copy number gain	18	MDM2, HOXC11, ALDH2, ARID2, BCL7A, WIF1, KMT2D, PTPN11, BTG1, LRIG3, HMGA2, HOXC13, NACA, CDK4, KRAS, ATF1, DDIT3, SH2B3
14:19,377,711-92,058,289 q11.2-q32.12	broad copy number gain	7	NKX2-1, CCNB1IP1, KTN1, GPHN, MAX, TSHR, NIN
17:29,483,070-29,685,567 q11.2	focal copy number gain; partial	1	NF1
17:41,247,899-41,342,646 q21.31	focal amplification; partial	1	BRCA1
18:48,265-78,005,193 p11.1-q23	broad copy number gain	5	MALT1, SS18, BCL2, ZNF521, SETBP1
2:105,858,873-136,590,718 q12.1-q21.3	broad copy number loss	3	PAX8, TTL, ERCC3
2:176,945,198-177,054,701 q31.1	focal copy number loss	2	HOXD11, HOXD13
3:108,308,169-197,894,642 q13.13-q29	broad copy number gain	13	SOX2, ETV5, RPN1, BCL6, MLF1, LPP, FOXL2, WWTR1, TFRC, GATA2, PIK3CA, EIF4A2, GMPS
3:239,468-56,655,601 p14.3-p26.3	broad copy number gain	12	FANCD2, PPARG, MYD88, SRGAP3, XPC, PBRM1, VHL, MLH1, CTNNB1, SETD2, RAF1, BAP1
3:56,707,678-108,288,343 p11.1-q13.13	broad copy number gain	5	MITF, FHIT, CBLB, TFG, FOXP1
7:100,682,784-156,802,697 q22.1-q36.3	broad copy number gain	7	EZH2, POT1, BRAF, SMO, KIAA1549, MET, CREB3L2
7:6,713,827-100,663,455 p11.1-q22.1	broad copy number gain	13	EGFR, HOXA11, CDK6, HOXA13, SBDS, HOXA9, ELN, JAZF1, AKAP9, HIP1, HNRNPA2B1, ETV1, IKZF1
8:17,507,411-18,666,278 p22	focal amplification	1	PCM1
8:27,685,666-38,965,271 p11.22-p21.1	broad copy number gain	3	WRN, FGFR1, WHSC1L1
8:39,785,524-142,190,897 p11.1-q24.3	broad amplification	10	MYC, NDRG1, COX6C, HOOK3, EXT1, CHCHD7, HEY1, TCEA1, PLAG1, NCOA2
