# Supplementary material for: Molecular medicine tumor board: whole-genome sequencing to inform on personalized medicine for a man with advanced prostate cancer
Source: Prostate Cancer Prostatic Dis. 2021 Feb 10;24(3):786–93. doi: 10.1038/s41391-021-00324-5 (PMC8384621; doi:10.1038/s41391-021-00324-5)
Supplement: Supplementary file 2 — Supplementary Table 2 [file 41391_2021_324_MOESM2_ESM.pdf]

Gene name	Classification	Reference Allele	Tumor Allele 1	Tumor Allele 2	AA change	Tumor (Normal) read depth	Tumor VAF
VILL 3:38,040,913	missense	G	G	A	p.G389S	220 (191)	33.6%
RSL1D1 16:11,931,711	missense	G	G	A	p.S469F	187 (162)	22.46%
SLC14A2 18:43,204,633	missense	T	T	C	p.S2P	224 (175)	25.89%
SH3BP4 2:235,950,877	missense	C	C	G	p.F488L	92 (66)	13.04%
NLRP5 19:56,565,117	missense	G	G	C	p.G1081A	109 (231)	23.85%
FLRT3 20:14,307,329	missense	C	C	T	p.R275Q	86 (60)	31.40%
LIPI 21:15,516,948	missense	C	C	T	p.E452K	64 (30)	59.4%
CCNT2 2:135,676,515	missense	G	G	C	p.D31H	97 (449)	21.65%
TEP1 14:20,841,707	missense	C	C	T	p.V2214I	40 (43)	12.50%
CCDC114 19:48,801,561	missense	C	C	T	p.S389N	238 (116)	30.25%
THNSL2 2:88,474,888	missense	C	C	T	p.T180M	171 (268)	16.37%
SORCS3 10:106,960,921	missense	G	G	A	p.R724H	154 (127)	22.08%
AP1M2 19:10,692,452	nonsense	G	G	A	p.Q124X	60 (39)	10.00%
MAMLD1 X:149,638,875	missense	C	C	A	p.P344T	40 (30)	15.00%
CSMD1 8:3,245,030	missense	A	A	G	p.L923S	167 (191)	43.11%
AGT 1:230,841,854	missense	C	C	T	p.G317S	148 (244)	25.00%
DGKH 13:42,729,863	missense	G	G	A	p.A184T	151 (96)	19.87%
VAV2 9:136,633,640	missense	C	C	T	p.R838Q	182 (62)	10.44%
