# Supplementary material for: Molecular medicine tumor board: whole-genome sequencing to inform on personalized medicine for a man with advanced prostate cancer
Source: Prostate Cancer Prostatic Dis. 2021 Feb 10;24(3):786–93. doi: 10.1038/s41391-021-00324-5 (PMC8384621; doi:10.1038/s41391-021-00324-5)
Supplement: Supplementary file 3 — Supplementary Table 3 [file 41391_2021_324_MOESM3_ESM.pdf]

Gene	Aberration in subject's genome	Aberration in PCAWG cohort	PCAWG cohort Frequency
MET	Coding	nonsynonymous SNV	1/199
SPOP	Coding	nonsynonymous SNV	15/199
CCNT2	Coding	NA	NA
MAFB	Coding	NA	NA
TNFAIP3	Non-coding	synonymous SNV<br>non-coding non-HFI SNV	1/199<br>1/199
GOPC	Non-coding	nonsynonymous SNV<br>non-coding HFI SNV	2/199<br>7/199
PAX5	Non-coding	non-coding HFI SNV	6/199
GATA3	Non-coding	NA	NA
BATF	Non-coding	NA	NA
BRF1	Non-coding	non-coding non-HFI SNV	8/199
